# Transcription analysis on response of porcine alveolar macrophages to *Haemophilus parasuis*

**DOI:** 10.1186/1471-2164-13-68

**Published:** 2012-02-13

**Authors:** Yang Wang, Chong Liu, Ying Fang, Xiaoli Liu, Wentao Li, Shuqing Liu, Yingyu Liu, Yuxi Liu, Catherine Charreyre, Jean-Christophe Audonnet, Pin Chen, Qigai He

**Affiliations:** 1 State key Laboratory of Agricultural Microbiology, Division of Animal Infectious Disease, Huazhong Agricultural University, Wuhan, Hubei, China; 2 Merial SAS, Lyon, France; 3 Keqian Animal Biological Products Co., Ltd, China

## Abstract

**Background:**

*Haemophilus parasuis *(*H. parasuis*) is the etiological agent of Glässer's disease in pigs. Currently, the molecular basis of this infection is largely unknown. The innate immune response is the first line of defense against the infectious disease. Systematical analysis on host innate immune response to the infection is important for understanding the pathogenesis of the infectious microorganisms.

**Results:**

A total of 428 differentially expressed (DE) genes were identified in the porcine alveolar macrophages (PAMs) 6 days after *H. parasuis *infection. These genes were principally related to inflammatory response, immune response, microtubule polymerization, regulation of transcript and signal transduction. Through the pathway analysis, the significant pathways mainly concerned with cell adhesion molecules, cytokine-cytokine receptor interaction, complement and coagulation cascades, toll-like receptor signaling pathway, MAPK signaling pathway, suggesting that the host took different strategies to activate immune and inflammatory response upon *H. parasuis *infection. The global interactions network and two subnetworks of the proteins encoded by DE genes were analyzed by using STRING. Further immunostimulation analysis indicated that mRNA levels of S100 calcium-binding protein A4 (S100A4) and S100 calcium-binding protein A6 (S100A6) in porcine PK-15 cells increased within 48 h and were sustained after administration of lipopolysaccharide (LPS) and Poly (I:C) respectively. The *s100a4 *and *s100a6 *genes were found to be up-regulated significantly in lungs, spleen and lymph nodes in *H. parasuis *infected pigs. We firstly cloned and sequenced the porcine *coronin1a *gene. Phylogenetic analysis showed that poCORONIN 1A belonged to the group containing the *Bos taurus *sequence. Structural analysis indicated that the poCORONIN 1A contained putative domains of Trp-Asp (WD) repeats signature, Trp-Asp (WD) repeats profile and Trp-Asp (WD) repeats circular profile at the N-terminus.

**Conclusions:**

Our present study is the first one focusing on the response of porcine alveolar macrophages to *H. parasuis*. Our data demonstrate a series of genes are activated upon *H. parasuis *infection. The observed gene expression profile could help screening the potential host agents for reducing the prevalence of *H. parasuis *and further understanding the molecular pathogenesis associated with *H. parasuis *infection in pigs.

## Background

The pig is an important agricultural animal and is an excellent mammalian model for biomedical research [1,2]. *H. parasuis *is the etiological agent of porcine polyserositis and arthritis (Glässer's disease) characterized by fibrinous polyserositis, meningitis and polyarthritis, causing severe economic losses to the swine industry [3]. To date, 15 serovars of *H. parasuis *have been identified [4]. *H. parasuis *infection can be acute or chronic, depending on the immunological status of the herd [3]. The infection by *H. parasuis *has become an increasing threat to early-weaned pigs and in pig herds of high health status [5,6].

The innate immune response in vertebrates is the first defense line against invading microorganisms. The main players in innate immunity are phagocytes such as neutrophils, dendritic cells and macrophages [7]. As a major component of the host innate immunity, macrophages have essential roles in host defense to infection, because they often mediate the killing of microbes as well as initiate, maintain and resolve host inflammatory responses by releasing cytokines and chemokines [8-11]. Bacterial pathogens that overcome host defenses ensure their ability to survive and propagate [10,12]. The diversity of bacteria and the differences in their pathogenesis may lead to pathogen-specific responses of macrophages [10]. A greater understanding of the complex interactions, which occur between the macrophages and pathogen, could lead to the identification of the host defense strategies and the complementary pathogen evasion strategies [8,10]. The interactions between *H. parasuis *with porcine alveolar macrophages have been studied [13], but the detailed mechanisms of how porcine alveolar macrophages response to *H. parasuis *infection are not well elucidated. The high throughput cDNA microarray represents a powerful tool for analyzing the molecular events in bacteria-host cell interactions [14]. This technology has been useful in identifying changes in gene expression both in cultured cells and in whole organisms infected with pathogens [12,15,16]. In this study, we applied this high throughput cDNA microarray assay to improve our understanding of the innate immune response of macrophages to *H. parasuis *infection.

## Results

### Clinical evaluation of infected pigs

In the challenge group, at 144 h post-infection, all three pigs had a rectal temperature of over 40.6°C and displayed lateral recumbency or labored breathing. At 144 h post-infection, all pigs including three control pigs were euthanized by intravenous administration of an overdose of sodium pentobarbital. In the challenge group, severe fibrinous polyserositis, arthritis and meningitis were observed at necropsy. On the other hand, in the control group, three pigs remained clinically normal throughout the experiment and did not have lesions at necropsy. The detection of *H. parasuis *by bacterial isolation, nested PCR and LAMP in different samples are shown in Table 1. The results indicated that the *H. parasuis *could be detected in the lymph nodes, lungs and spleen in all of the three pigs that challenged with *H. parasuis *serovar 5 SH0165 strain. In contrast, *H. parasuis *could not be detected by the three methods in the control group.

**Table 1:** Results of culture, nested PCR and LAMP analysis for three pigs challenged with *H. parasuis *serovar 5, presented as the number of pigs positive/pigs samples

Samples	***H. parasuis *culture**^**a**^	Nested PCR	LAMP
**Brain**	0/3	1/3	1/3
**Lymph node**	3/3	3/3	3/3
**Tonsil**	2/3	2/3	2/3
**Lung**	3/3	3/3	3/3
**Pericardial fluid**	2/3	2/3	2/3
**Heart**	1/3	1/3	1/3
**Spleen**	3/3	3/3	3/3
**Total site**	14/21	15/21	15/21
**%positive**	67	71	71

a: if *H. parasuis *was not identified by culture due to contaminated overgrowth, presence of *H. parasuis *was checked by nested PCR and LAMP on a colony sweep.

### Overview of differential expressed genes in PAMs

**Figure 1:**
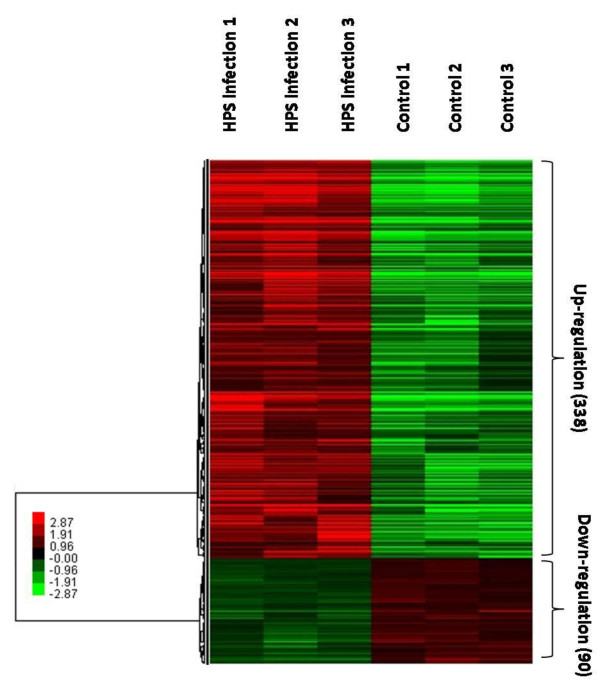
**A hierarchical cluster of 428 transcripts following *H. parasuis *infection in PAMs**. Each row represents a separate transcript and each column represents a separate piglet. Color legend is on the left, red indicates increased transcript expression levels, whereas green indicates decreased levels compared with normal samples. Up-/down-regulated response transcripts are highlighted on the right with the No. in each cluster in parentheses. Fold change is calculated based on the mean intensity value from 3 donors within each group.

To investigate the dynamic gene transcriptional profiles of PAMs in response to *H. parasuis *infection, six microarrays were used in this experiment, corresponding to the RNAs from PAMs of three *H. parasuis *infected piglets and three controls. The total RNA samples were hybridized with Affymetrix GeneChip Porcine Genome Array, and the microarray data were analyzed using Significance Analysis of Microarrays (SAM) [17]. Hybridization results indicated that 14,228 and 13,813 probes sets, corresponding to 58.9% and 57.3% of all probe sets, were detected in *H. parasuis *serovar 5 and mock-infected PAMs (Additional file 1). After quantile normalization and statistical analysis, 623 transcripts were identified at SAM |Score(d)| ≥ 2. Furthermore, Genes whose relative transcription levels showed a fold change FC ≥ 1.33 and SAM |Score(d)| ≥ 2 were considered to be up-regulated, and those with an FC ≤ 0.75 and SAM |Score(d)| ≥ 2 were considered to be down-regulated. In this study, 575 transcripts showed a level of expression that differed significantly from that of the control group with *H. parasuis *serovar 5 SH0165 strain infected group. These included 428 genes annotated with DAVID or by searching against the GenBank database (Additional file 2). Among these, 338 genes were up-regulated and 90 genes were down-regulated (Figure 1).

The functions of the DE genes were analyzed by Molecular Annotation System 3.0 software http://www.capitabio.com[18]. In the MAS 3.0 tool, the GO terms and KEGG pathways are ranked with statistical significance by calculating their *p*-values based on hypergeometric distribution [19]. Go terms and KEGG pathways with *p-*values less than 0.05 are considered statistically significant [20]. Among the 428 annotated genes, a total of 229 genes were grouped into 156 categories based on biological process Gene Ontology (GO) terms with the *p*-values less than 0.05 (Additional file 3). Several GO terms were associated with the immune system. These were "inflammatory response" (GO:0006954, *p *= 2.06E-04), "immune response" (GO:0006955, *p *= 1.75E-03), "complement activation, classical pathway" (GO:0006958, *p *= 9.48E-04) and "leukocyte migration" (GO:0050900, *p *= 9.55E-03). Particularly, the DE genes associated with immune and inflammatory response suggested that they play roles in host defense response to *H. parasuis *infection (Table 2). To gain insight into the different biological processes of *H. parasuis *infection, a pathway analysis by KEGG database was performed on DE genes (Additional file 4) [18]. The significant pathways mainly contained: cell adhesion molecules (*p *= 3.74E-12), cytokine-cytokine receptor interaction (*p *= 2.18E-10), complement and coagulation cascades (*p *= 1.16E-07), toll-like receptor signaling pathway (*p *= 1.93E-05), MAPK signaling pathway (*p *= 7.63E-04), which suggested that the host took different strategies to activate immune and inflammatory response upon *H. parasuis *infection.

**Table 2:** The DE genes associated with immune and inflammatory response in PAM after *H. parasuis *serovar 5 infection 6 days

Functional classification	Gene Description	GenBank ID	Fold Change	Score (d)	*q*-value (%)
**Immune and inflammatory response**					
	***Fc fragment of IgG, low affinity IIb, receptor***	NM_001033013.1	24.5846355	7.08397559608837	0
	***Chemokine (C-C motif) ligand 2***	NM_214214.1	10.66938955	3.61587781284878	0.542032509
	***Chemokine (C-C motif) receptor 5***	NM_001001618	9.336150413	6.3078994808823	0
	***Chemokine (C-C motif) ligand 3-like 1***	NM_001009579.1	3.984785551	5.9277826808052	0
	***Chemokine ligand 4***	NM_213779.1	3.27736509	4.8388236052463	0
	***S100 calcium-binding protein A14***	AK240199	1.774087863	2.21222644012883	3.76387374
	***S100 calcium-binding protein A6***	NM_001044557.1	2.496131662	2.1433599692719	4.658259579
	***S100 calcium-binding protein A4***	XM_001929560.1	5.120172033	9.15364933294775	0
	***CD247***	AK239043.1	2.33533278	3.72704398328664	0.542032509
	***CD14 molecule***	NM_001097445.2	2.798496395	3.06576353306702	0.993281247
	***low density lipoprotein receptor related protein 11***	NM_032832.5	6.960187733	5.253204788	0
	***chemokine (C-X-C motif) receptor 7***	XM_003133759.1	6.215116279	6.960762567	0
	***ring finger protein 128***	NM_001076071.1	14.61041884	4.192025143	0
	***transforming growth factor, beta-induced, 68 kDa***	XM_001111447.2	4.110924648	4.879669438	0
	***lymphotoxin beta***	AK234043.1	2.971296231	3.522685959	0.542032509
	***interleukin 1 beta***	NM_214055.1	2.897736981	2.601362002	2.021414468
	***chemokine (C X C motif) ligand 14***	XM_003123950.1	8.306998658	3.343782966	0.790729777
	***granzyme H***	NM_001143693.1	4.809267807	3.458161153	0.790729777
	***protein kinase C theta***	NM_001001640.1	3.106802289	3.332538502	0.790729777
	***superoxide dismutase 2, mitochondrial (SOD2)***	NM_214127.2	2.647298815	2.852910519	1.387404311
	***caveolin 2***	NM_001123091.1	5.305246137	4.935617987	0
	***CD2 molecule***	NM_213776.1	4.680082645	4.576351885	0

### Validation of microarray data by quantitative real-time PCR (qPCR)

In order to confirm the statistical significance of our findings, we performed quantitative real-time PCR (qPCR) analysis of the relevant genes in our original samples used in microarray study. Eleven genes were selected for qPCR analysis. Ten selected genes that were up-regulated in microarray also showed significantly higher expression in *H. parasuis *serovar 5 infected samples than in the control samples determined by qPCR analysis. The ppp1r13l gene that was down-regulated in microarray data also showed significantly lower expression in *H. parasuis *serovar 5 infected samples than in the control samples by qPCR (Table 3).

**Table 3:** Validation of microarray results by qPCR

Gene	Accession	Primers	Microarray fold change	qPCR fold change	*p*-value	Product size
***CD14 molecule***^***a***^	NM_213973.1	**F:**GCAGAGGCTTTGAGGACCTTATC	2.798496395	3.835	0.0001	154 bp
		**R:**GCTGCGGATGCGTGAAGTT				
***CD3e molecule, epsilon (CD3-TCR complex)^a^***	NM_214227.1	**F:**ACCTCTTAGTTCCTCCCTTTG	5.671628855	4.619	0.005	137 bp
		**R:**TGCCAGCATTTACCCAGTC				
***heat shock protein 70.2^b^***	NM_213766.1	**F:**AGGCGGAGAAGTACAAAGCG	3.374059696	5.775	0.007	257 bp
		**R:**GATGGGGTTACACACCTGCTC				
***TIMP metallopeptidase inhibitor 1^b^***	NM_213857.1	**F:**CGCCTCGTACCAGCGTTAT	8.614065715	11.357	0.008	127 bp
		**R:**GTGGAAGTATCCGCAGACGC				
***superoxide dismutase 2, mitochondrial (SOD2)^b^***	NM_214127.2	**F:**TCTGGACAAATCTGAGCCCT	2.647298815	2.1687	0.003	119 bp
		**R:**GACGGATACAGCGGTCAACTT				
***S100A4***	XM_001929560.1	**F:**GTCCACCTTCCACAAGTA	5.120172033	5.4957	0.006	152 bp
		**R:**TGTCCAAGTTGCTCATCA				
***S100A6***	NM_001044557.1	**F:**AAGGCTGATGGAAGACTT	3.082743701	5.5237	0.016	105 bp
		**R:**TTGAGGGCTTCATTGTAGA				
***Caveolin 1***	NM_214438.2	**F:**CTTCACCACCTTCACTGT	4.301157871	2.319	0.041	184 bp
		**R:**GGAATAGACACGGCTGAT				
***Caveolin 2***	NM_001123091.1	**F:**GCAGACAATATGGAAGAGTG	5.305246137	3.327	0.011	85 bp
		**R:**CAGGCTGACAGAAGAGAA				
***coronin, actinbinding protein, 1A (CORO1A)***	BT025463.1	**F:**GTGGACTGGAGCCGAGATGGA	6.065784814	5.7667	0.0001	200 bp
		**R:**GCCACCTGCCGCTCACTC				
***protein phosphatase1, regulatory (inhibitor) subunit 13 like (PPP1R13L)***	XM_002801296.1	**F:**CACCAGAGCAGCCGCAGAG	0.506516661	0.2116	0.0001	107 bp
		**R:**GTCCAGGAGGAGCACCAGAGG				

a: Primers from reference 87

b: Primers from reference 16

### STRING analysis of the relationships between DE genes

**Figure 2:**
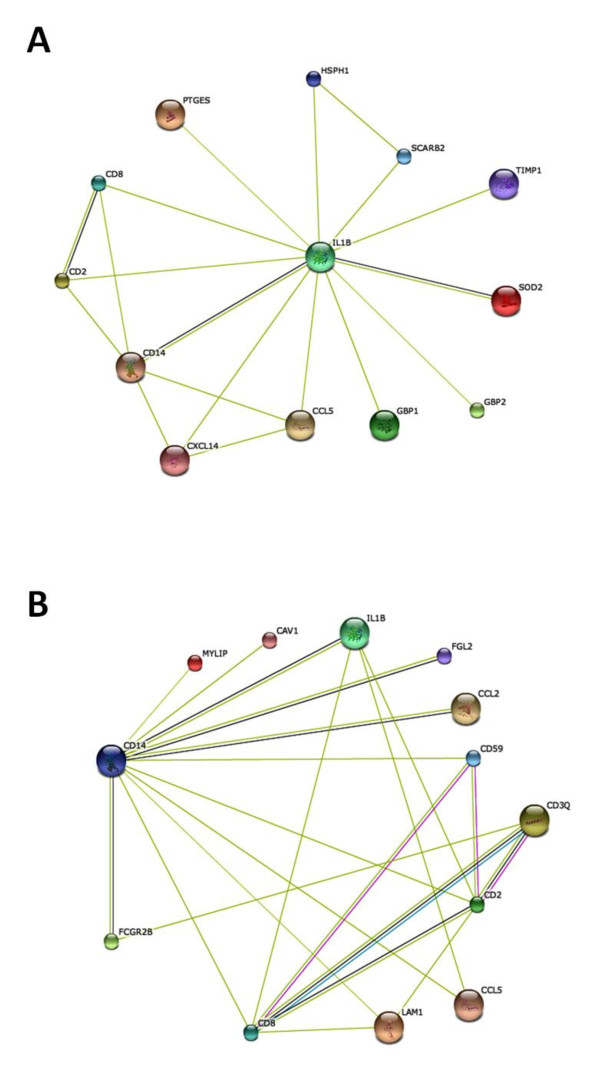
**STRING analysis of the relationship between DE genes**. The DE genes were analyzed using the *Sus Scrofa *STRING database. The network nodes represent the proteins encoded by the DE genes. Seven different colored link a number of nodes and represent seven types of evidence used in predicting associations. A red line indicates the presence of fusion evidence; a green line represents neighborhood evidence, a blue line represents coocurrence evidence; a purple line represents experimental evidence; a yellow line represents textmining evidence; a light blue line represents database evidence and a black line represents coexpression evidence. A: The network of DE genes related to IL-1β. B: The network of DE genes related to phagocytosis.

STRING is a web-based interface that could predict protein associations which can mean direct physical binding and can also mean indirect interaction such as participation in the same metabolic pathway or cellular process on the basis of genomic context, high-throughput experiments, co-expression and data from the literature http://string.embl.de[21,22]. DE genes were analyzed using STRING for predicting network of proteins encoded by DE genes. Among the 428 annotated DE genes, 236 genes containing 181 up-regulated genes and 55 down-regulated genes were eligible to STRING analyses when the *Sus Scrofa *database was chosen. In order to seek the possibility of the associations between DE genes, the combined score of 0.15 was chosen. The network of predicted associations for all of the DE genes encoded proteins are shown in Additional file 5. Some molecules are the key molecules that link to other proteins according to the STRING analysis. However, many proteins do not link to others, indicating that their functions are unrelated or unknown. As shown in Figure 2A, a total of 12 DE genes encoded proteins are associated with IL-1β according to the textmining evidence, and they form the IL-1β network. Furthermore, the CD14 and SOD2 are associated with IL-1β according to the co-expression evidence. Two of phagocytosis-related genes (*cd14, fcgr2β*) are associated with a total of 11 DE genes encoded proteins (Figure 2B) according to the textmining evidence, and they form the phagocytosis network. Furthermore, the IL-1β, FGL-2, CCL2 and FCGR2β are associated with CD14 according to the co-expression evidence.

### Identification of novel infection-related DE genes

In order to identify novel candidates for disease-related DE genes, we evaluated the DE genes that were not highlighted in the KEGG or STRING analysis. Although the *s100a4, s100a6 *and *coronin 1a *were not highlighted in the KEGG or STRING analysis, they were found to play roles in the immune response [23-34]. These observations suggest that the three genes may be novel candidates for disease-related DE genes.

Among the three genes, *coronin 1a *has not been identified in pigs before. So we cloned and sequenced the porcine *coronin 1a *gene according to description of Liu et al [35] and the sequence was submitted to the GenBank [GenBank: JN092377]. The full-length cDNA of porcine *coronin 1a *contains 1386 bp and 461 amino acid residues. Multiple sequence alignment with the identified *coronin 1a *of cattle, human, mouse, rat and the predicted *coronin 1a *of other species showed that the nucleotide sequence of the poCORONIN 1A ORF is 93.58%, 93.58%, 92.42%, 92.35%, 92.28%, 91.70%, 87.81% and 86.87% identical to that of panda, cattle, human, chimpanzee, northern white-cheeked gibbon, common marmoset, mouse and rat *coronin 1a*, respectively. At the amino acid level, the corresponding identities were 96.96%, 97.61%, 95.66%, 95.66%, 95.88%, 96.10%, 93.71% and 92.84%, respectively. To define the molecular evolutionary history of poCORONIN 1A, protein sequences from 9 vertebrates were obtained to construct a phylogenetic tree. Phylogenetic analysis showed that poCORONIN 1A belongs to the group containing the *Bos taurus *sequence (Additional file 6). Structural analysis with the ExPASy server http://expasy.org/ indicated that the poCORONIN 1A contains putative domains of Trp-Asp (WD) repeats signature, Trp-Asp (WD) repeats profile and Trp-Asp (WD) repeats circular profile at the N-terminus (Additional file 7).

### Expression analyses of S100A4, S100A6 in PK15 cells stimulated with LPS and Poly (I:C)

In order to investigate the expression patterns of *s100a4 *and *s100a6 *under general conditions that mimic bacterial and viral infection, the immunostimulation assay was carried out in PK-15 cells by using the LPS and Poly (I:C) as the stimulators.

**Figure 3:**
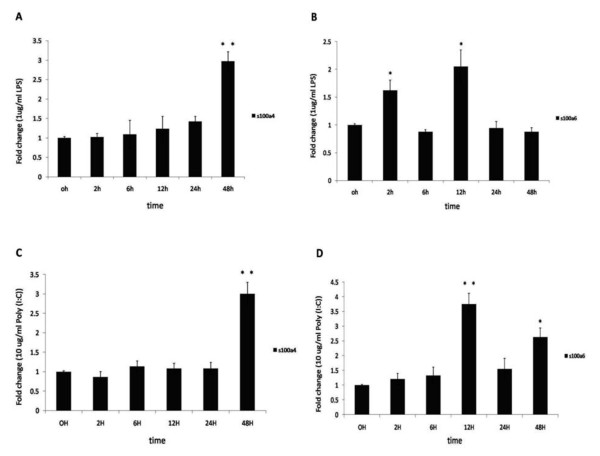
**Kinetic immune stimuli analyses challenged by LPS and Poly (I:C) in PK-15 cells**. A, B: LPS-induced expression of porcine *s100a4 *and *s100a6 *in PK-15 cells respectively. PK-15 cells were cultured with 1 μg/mL LPS for 48 h. C, D: Poly (I:C) induced expression of porcine *s100a4 *and *s100a6 *in PK-15 cells respectively. PK-15 cells were cultured with 10 μg/mL Poly (I:C) for 48 h. Relative expression of *s100a4 *and *s100a6 *were detected by qPCR and normalized to the expression of GAPDH. The fold increase is expressed as the mean of three replicates with SEM by comparison with the control (0 h). QPCR was performed using primers described in Table 3. The significance of difference for the expression compared to the untreated control (0 h) was calculated using two-directional paired Student's T-test. ** p ≤ 0.01; * p ≤ 0.05.

Overnight cultures of PK-15 cells were treated with 1 μg/ml LPS or 10 μg/ml Poly (I:C) for 0, 2, 6, 12, 24 and 48 h. LPS and Poly (I:C) stimulation did not induce expression of porcine *s100a4 *until 48 h (Figure 3A, C). LPS stimulation induced expression of *s100a6 *at 2 h and 12 h, after which *s100a6 *expression dropped and plateaued for 24-48 h (Figure 3B). After Poly (I:C) stimulation, the expression of *s100a6 *reached the peak at 12 h, after which *s100a6 *expression dropped at 24 h, and the up-regulation of *s100a6 *was again observed at 48 h (Figure 3D). These observations indicate that both LPS and Poly (I:C) can induce the expression of porcine *s100a4 *and *s100a6 *in vitro.

### In vivo expression of *s100a4 *and *s100a6 *in pigs with systemic infection of *H. parasuis*

**Figure 4:**
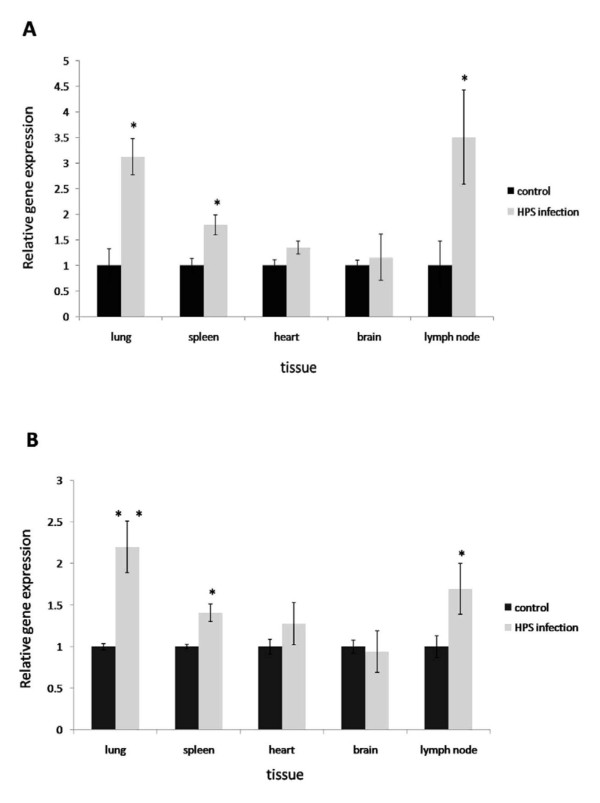
**Quantitative expression of *s100a4 *and *s100a6 *in five tissues from pigs with Glässer's disease**. A: Increased in vivo gene expression of *s100a4 *in lungs, spleen, lymph nodes of pigs with Glässer's disease. B: Increased in vivo gene expression of *s100a6 *in lungs, spleen, lymph nodes of pigs with Glässer's disease. Relative expression of *s100a4 *and *s100a6 *were detected by qPCR and normalized to the expression of GAPDH. The fold increase is expressed as the mean of three replicates with SEM by comparison with the control. The significance of difference for the expression compared to the control was calculated using two-directional paired Student's T-test. ** p ≤ 0.01; * p ≤ 0.05.

In order to understand the expression of the *s100a4 *and *s100a6 *in pigs with systemic infection of *H. parasuis*, the different tissues obtained from the *H. parasuis *infected pigs and the controls were selected for the qPCR analysis. Our qPCR examination demonstrated that the increasing expression of *s100a4 *was observed in the lungs, spleen and lymph nodes of pigs infected with *H. parasuis *for 6 days (Figure 4A). The expression of *s100a6 *in the lungs, spleen and lymph nodes had the same expression tendencies (Figure 4B). However, in brain and heart of *H. parasuis *infected pigs, the expression of *s100a4 *and *s100a6 *did not show significant changes compared to the controls.

## Discussion

During infection, *H. parasuis *has to reach the lung and survive the host pulmonary defenses before invading the blood stream [13]. In the lung, bacteria have to confront alveolar macrophages, whose main roles include: ingestion of bacteria by phagocytosis, destruction of bacteria within phagolysosomes and recruitment of inflammatory cells to the site of infection via chemokines and acute-phase proteins [36].

Phagocytosis is a cytoskeleton-dependent process of engulfment of large particles, and macrophages could present a restricted number of phagocytic receptors that induce rearrangements in the actin cytoskeleton that lead to the internalization of the particle [36]. Phagocytosis is a key mechanism used by macrophages to control virulent Pasteurellaceae, such as *Pasteurella multocida, Haemophilus parasuis, Haemophilus influenzae, Actinobacillus pleuropneumoniae *[13,37-40]. In this study, the *cd14 *[41-44], *hmox1 *[45], *fcgr2β *[46] and *abca1 *[47] genes, which were identified as DE genes, were also found to be involved in the phagocytosis. Meanwhile, the STRING analysis indicated that many DE gene encoded proteins could interact with CD14 and FCGR2β molecules, suggesting that PAMs may upregulate these genes to facilitate the phagocytosis of *H. parasuis *or other cells to play their immunological roles.

One of the important mechanisms used by macrophage to play its immunological functions is to kill bacteria by the activation and recruitment of antibacterial effectors to the phagolysosome [36]. The fusion of phagosomes with lysosomes results in the formation of phagolysosomes [11,36]. In our study, we found two DE genes that were related to the formation of phagolysosome, that is, *smpd1 *[48] and *coronin 1a *[29-31]. Interestingly, many groups have reported that the CORONIN 1A could prevent lysosomal delivery and allow the bacteria to survive intracellularly [30,32-34]. In the course of infection, *H. parasuis *has to survive from the host pulmonary defense, such as alveolar macrophages, to produce disease. In this way, the up-regulation of *coronin 1a *gene may facilitate the *H. parasuis *in producing the disease.

Interleukin-1 beta (IL-1β) is an important inflammation-associated gene that is up-regulated in many microarray experiments [11,14,16,49]. Interestingly, Wilkinson et al reported that an increase in IL-1β gene expression is observed in *H. parasuis-*infected lungs [50]. In our study, IL-1β was also up-regulated in *H. parasuis-*infected PAMs. Unsurprisingly, STRING analysis also revealed that many molecules encoded by up-regulated genes interact with IL-1β and form the IL-1β network. Meanwhile, the pathway analysis indicated that IL-1β is in some pathways, such as cytokine-cytokine receptor interaction (*p *= 2.18E-10), MAPK signaling pathway (*p *= 7.63E-04), and toll-like receptor signaling pathway (*p *= 1.93E-05). CCL5/RANTES plays an important role in regulating the movements of inflammatory cells to the infection sites [51,52]. Many viruses, such as Japanese encephalitis virus (JEV) [53], respiratory syncytial virus (RSV) [54], influenza virus A [55] and porcine reproductive and respiratory syndrome virus (PRRSV) [51] have been shown to induce CCL5. In addition, some papers have reported that the CCL5 could be induced in macrophages by bacterial infections, such as *Salmonella typhimurium *infection, *Streptococcus pyogenes *infection and *Lactobacillus rhamnosus *infection [56,57]. Interestingly, in our study, the up-regulation of CCL5 was observed in *H. parasuis*-infected PAMs, which suggested that CCL5 plays a role in the host response against *H. parasuis *infection. Thus, during the *H. parasuis *infection, the PAMs mount a powerful inflammatory response in an effort to clear this pathogen. Alternatively, the influx of inflammatory cells to the site of infection may provide additional host cells for *H. parasuis *infection. However, sustained or excessive production of inflammatory cytokines can have damaging consequences. To counterbalance inflammatory cytokines, anti-inflammatory cytokines are produced. Anti-inflammatory cytokines include interleukin 10 (IL-10), transforming growth factor β (TGF-β), and IL-1 receptor antagonist (IL-1RA) [11,58,59]. Wilkinson et al reported that the IL-1β and its antagonist, IL-1RA are both more highly expressed in "susceptible" animals challenged with *H. parasuis *[50]. In our study, TGF-β, an anti-inflammatory cytokine, was increased in *H. parasuis *infection group. During *H. parasuis *infection, anti-inflammatory signals may decrease the potentially damaging effects of proinflammatory cytokines on host tissue.

Macrophage also effectively controls bacterial infection by producing of reactive species such as oxygen species and nitric oxide (NO). Sustained production of NO endows macrophages with cytostatic or cytotoxic activity against viruses, bacteria, fungi, protozoa, helminths and tumor cells. Unsurprisingly, *H. parasuis *infection could cause up-regulated expression of a large set of genes involved in the nitric oxide production. These genes were: *spr, rora, klrk1, sod2 *and *il-1β *[60-66]. The up-regulated genes related to the nitric oxide production may contribute to the PAM for confronting *H. parasuis *infection.

The DE genes that are related to phagocytosis, formation of phagolysosome, chemokines production, and nitric oxide production may help us to better understand the complicated mechanisms by which PAMs play their functions. Another highlight of our study is the new identified candidate genes that may be implicated in the pathogenesis of Glässer's disease. These genes could help to screen the potential host agents for reducing the prevalence of *H. parasuis *and further understand the molecular pathogenesis associated with *H. parasuis *infection in pigs. These genes are: *s100a4, s100a6, caveolin 2 *and *ppp1r13l*.

S100 A4 and S100 A6 belong to the S100 family that contained 2 EF-hand calcium-binding motifs [23,27]. Two of S100 family genes (S100 calcium-binding protein A8 and A9) are dramatically up-regulated in spleen and lung following *H. parasuis *infection [67]. Meanwhile, many other S100 family genes are up-regulated following different bacterial and viral infection [16,18,67,68], suggesting that the S100 family genes play roles in the immune response to infections. In our study, the S100 calcium-binding protein A4 and A6 were up-regulated after *H. parasuis *infection when determined by microarray and qPCR. Further immunostimulation analysis indicated that the mRNA levels of S100 calcium-binding protein A4 (S100A4) and S100 calcium-binding protein A6 (S100A6) in porcine PK-15 cells increased within 48 h and were sustained after administration of LPS and Poly (I:C) respectively. We also found that the *s100a4 *and *s100a6 *genes were up-regulated in lungs, spleen and lymph nodes in *H. parasuis *infected pigs. Interestingly, the kidney fibrosis [24-26,69], liver fibrosis [70,71], lung fibrosis [72-74], cardiac fibrosis [23,75,76] and peritoneal fibrosis [77] are found to be related to the expression of *s100a4*. Glässer's disease is characterized mainly by fibrinous polyserositis, meningitis, and arthritis. In this way, we hypothesized that the increase expression of *s100a4 *may underlie fibrosis during *H. parasuis *infection in pigs. Meanwhile, some reports indicated that *s100a6 *plays roles in cell proliferation and signaling transduction [27,28]. Therefore, the *s100a4 *and *s100a6 *genes could be two novel genes related to *H. parasuis *infection.

Caveolins are the major components and protein markers of caveolae that are 50-100 nm invaginations of membrane. The caveolin gene family includes three members in vertebrates, caveolin-1, caveolin-2 and caveolin-3, of which caveolin 1 and caveolin 2 have been detected in mouse macrophages [35,78]. Caveolin 1 molecule is related to *H. parasuis *infection [35]. Caveolin 2, which localizes to the Golgi complex but redistributes to plasma membrane, caveolae and rafts when co-expressed with caveolin 1, is a potential key molecule related to the *Pseudomonas *infection causing pneumonia in patients with cystic fibrosis and other immunocompromising conditions [79,80]. In our study, the *caveolin 2 *gene was highly expressed in PAM isolated from the *H. parasuis *serovar 5 challenged group. Therefore, in addition to the *caveolin 1 *gene, the *caveolin 2 *gene may be a novel candidate gene related to *H. parasuis *infection.

The NF-kappa B (NF-κB) signaling pathway is important in signal transduction during the innate immune response [36]. NF-κB signaling relies on the targeting of IκB (inhibitor of NF-κB) subunit to the proteasome to allow NF-κB to translocate from the cytosol to the nucleus where it activates gene transcription [81]. The PPP1R13L is mentioned as a novel inhibitor of NF-κB [82]. In our study, microarray and qPCR analysis indicated that the mRNA of PPP1R13L was down-regulated significantly compared to control. The IPA network indicated that the PPP1R13L could directly or indirectly interacts with many molecules, such as micro RNAs, transcriptions, enzymes, and cytokines (Additional file 8), suggesting that *ppp1r13l *gene is an innate immune related gene that plays a role in PAM during *H. parasuis *infection. The detailed mechanism of *ppp1r13l *gene in NF-κB signaling pathway in *H. parasuis *infected PAM needs further studies.

## Conclusion

This is the first study focusing on response of porcine alveolar macrophages to *Haemophilus parasuis *by using the Affymetrix GeneChip Porcine Genome Array. Although great efforts have been made to understand the molecular basis of *H. parasuis *infection, the cellular response to *H. parasuis *infection is still largely unknown. The high-density cDNA array technology to analysis of *H. parasuis*-infected PAM could improve our understanding of the *H. parasuis *infection. Our data show that a series of genes are activated upon *H. parasuis *infection. These genes are involved in inflammatory response, immune response, microtubule polymerization, regulation of transcript and signal transduction. Particularly, some genes related to phagocytosis, formation of phagolysosome, chemokines production and nitric oxide production could contribute to explain the complicated mechanisms by which PAM played its functions. Some new identified genes may also provide implication on the pathogenesis of Glässer's disease caused by *H. parasuis*.

## Methods

### Animals for Microarray experiment and porcine alveolar macrophages isolation

All animals' tissue collection procedures were performed according to protocols approved by the Hubei Province PR China for Biological Studies Animal Care and Use Committee. Six piglets which were obtained from a commercial herd free of Glässer's disease were weaned at 27 days, shipped to the Animal Disease Center of Huazhong Agricultural University, and raised with isolation facilities. Three piglets were randomly allocated to the non infected group and three to the infected group. The three piglets were intratracheally challenged with *H. parasuis *strain 0165 (serovar 5) at a dose of 6 × 10^9 ^colony-forming units (CFU). The noninfected group piglets were treated similarly with identical volume of PBS served as control. All piglets were determined to the HPS-free by serum indirect haemagglutination (IHA) test before artificial bacterial challenges. Clinical signs and lesions of Glässer's disease were apparent in the challenged group at 6 days post-infection (dpi). All piglets were slaughtered at 6 dpi. Bacterial isolation, nested PCR and LAMP were performed after the piglets were killed at 6 dpi. PAMs were isolated according to Olvera's description [13]. Briefly, Bronchoalveolar lavage of the lungs was performed with 100 mL aliquots of sterile PBS containing gentamicin at 70 μg/mL (Sigma-Aldrich). To collect the porcine alveolar macrophages (PAM), lavage fluids were centrifuged at 230 g for 15 min, and then cells were washed twice with Dulbecco's Modified Eagle's Medium (DMEM) with gentamicin (50 μg/mL). PAM isolation was confirmed by detection of macrophage markers (SWC3, CD169 and SLAII) in the cells by flow cytometry.

### RNA preparation for Microarray experiment

Total RNA were extracted from PAM of each group with Trizol (Invitrogen) then quantified using the NanoDrop 1000 Spectrophotometer (Thermo Fisher Scientific Inc., USA). The quality of the RNA was checked by formaldehyde denaturing gel electrophoresis in 1.2% agarose gels, which showed dispersed bands (28S and 18S) without any obvious smearing patterns that would indicate degradation.

### Microarray hybridization and data analyses

Affymetrix GeneChip Porcine Genome Array, which contains 24,123 probe sets to interrogate 23,256 transcripts in pig, represents 20,201 genes, was used in microarray analysis. Hybridization, data capture and analysis were performed by CapitalBio Corporation (Beijing, China), a service provider authorized by Affymetrix Inc. (Santa Clara, CA). Briefly, a total of 1 μg RNA was used for cDNA synthesis and to produce biotin-tagged cRNA with GeneChip IVT Labeling kit (Affymetrix). A total of 15 μg fragmented cRNA, with contol oligo B2 and eukaryotic hybridization controls (bioB, bioC, bioD, cre) was hybridized to each GeneChip array at 45°C for 16 hours (Affymetrix Gene Chip Hybridization Oven 640) according to manufacturer's instructions. After hybridization, the GeneChip arrays were washed and stained with streptavidin phycoerythrin onan (SAPE) with Affymetrix Fluidics Station 450 followed by scanning with the Affymetrix GeneChip Scanner 3000. Six microarrays were used in the experiment, corresponding to the RNAs from PAMs of three *H. parasuis *infected piglets and three controls.

The hybridization data were analyzed using GeneChip Operating Software (GCOS, version 1.4), which uses statistical criteria to generate a 'present' or 'absent' call for genes represented by each probe set on the array. The scanned images were first assessed by visual inspection and then analyzed to generate raw data files saved as CEL files using the default setting of GCOS 1.4. Microarray data were normalized using the robust multi-array average (RMA) method [83], which consists of three steps: background correction, quantile normalization (each performed at the individual probe level), and robust linear model fit using log-transformed intensities (at the probe set level). Significance Analysis of Microarrays (SAM) add-in to Microsoft Excel was used for comparisons of replicate array experiments. SAM identifies genes with statistically significant changes in expression by assimilating a set of gene-specific *t*-tests, and provides an estimate of the false discovery rate (FDR) from randomly generated data. Genes with scores higher than a threshold value or genes with FDR value lower than the threshold value were deemed potentially significant. Furthermore, fold-change analysis which calculates the ratios of geometric means of expression intensities of *H. parasuis*-infected PAMs relative to controls was performed. These ratios were reported as the up-or down-fold change. In this study, genes were considered statistically significant if they had SAM |Score(d)| ≥ 2 [84,85] and exhibited a fold change ≥ 1.33 and ≤ 0.75. DE genes performed for hierarchical cluster (Ver.3.0) and TreeView (Ver.1.1.1) analyses. Genes with significant similarities to the transcripts in nr database based on BLASTX searches were selected for GO analysis, performed by MAS 3.0 software which was based on DAVID database (CapitalBio, Beijing, China) [16]. Annotation results were obtained by inputting the list of gene symbol as identifier [18]. The Pathway analysis was done using the MAS 3.0 software which was based on the Kyoto Encyclopedia of Genes and Genomes database (KEGG) (CapitalBio, Beijing, China). All microarray results from this study were deposited in NCBI'S Gene Expression Omnibus (GEO) database, accession numbers are: Platform, GPL 3533, Samples, GSM 747145, GSM 747146, GSM 747147, GSM, 747148, GSM 747149, GSM 747150 with the series accession number GSE 30172.

### QPCR analysis

Total RNA were extracted from the PAMs of each group with Trizol (Invitrogen) and 5 μg of total RNA were used for first strand cDNA synthesis by using Superscript II cDNA amplification System (Invitrogen) following manufacturer's instructions. Real-time PCR was performed using LightCycler 480 (Roche Applied Science) and Quantitect SYBR Green PCR kit (Roche) following the companies' instructions. Briefly, PCR assay was performed under the following conditions: 95°C for 15 sec, 55°C for 15 sec and 72°C for 15 sec. Real-time PCR primers for each gene were indicated in Table 3. All the primers were originally designed using Primer 3 software (Rozen & Skaletsky, 2000) or according to the published papers. Results were calculated by minus delta delta threshold cycle (-ddCt) method. Briefly, the threshold cycle Ct_1 _of each sample reaction were deducted with the threshold cycle Ct_2 _of GAPDH reaction for normalization, then deducted from the threshold cycle Ct_3 _of calibration control (40 Cycles in this experiment); thus, the final result was represented by the formula: Ct_3_-(Ct_1_-Ct_2_).

### Expression of S100A4, S100A6 in PK-15 cells stimulated with LPS and Poly (I:C)

PK-15 cells have been shown especially useful for the study of infectious disease processes in swine [86,87]. In this study, 12 groups (with three repeats in each group, ~1 × 10^5 ^cells/samples) of PK-15 cells were grown in culture medium (DMEM) supplemented with 10% heat-inactivated fetal bovine serum at 37°C with 5% CO_2_. Adherent PK-15 cells were obtained by washing off nonadherent cells with warm culture medium and PBS twice, respectively. Adherent cells were further cultured in DMEM (control samples) or treated with 1 μg/mL LPS (Sigma-Aldrich, *E.coli *0127:B8) or 10 μg/mL Poly (I:C) (Sigma-Aldrich) respectively (stimulated samples) for 0 h, 2 h, 6 h, 12 h, 24 h and 48 h. Cells were harvested and total RNA were extracted as described above.

### DNA preparation from bacterial isolates and clinical samples

Bacterial cultures were harvested from trypticase soy agar (TSA) using an inoculation loop and were placed into a 1.5 mL tube to which was added with 500 μL of phosphate buffered saline (PBS). One milliliter of the fluid and 0.5 g of the tissue samples were respectively placed in sterile tubes containing 5 mL of trypticase soy broth (TSB), 5 μL nicotinamide adenine dinucleotide (NAD) and 500 μL sterilized fetal bovine serum and then incubated for 8 h at 37°C with agitation. Five hundred microliters of the suspension was removed to a new 1.5 mL tube. Tubes containing bacteria, tissue and fluid suspensions were centrifuged at 13,400 g for 5 min. After centrifugation, the supernatant was discarded and the remaining pellet was suspended in 200 μL PBS, boiled for 10 min. After boiling, tubes were centrifuged at 13,400 g for 5 min. Fifty microliters of supernatant from each sample containing extracted DNA were mixed with 50 μL of Tris-EDTA buffer and stored at 4°C. This final solution was used as DNA template in nested PCR and LAMP reaction. The primers for nested PCR and LAMP were listed in Additional file 9. The procedure of bacterial isolation, nested PCR and LAMP were carried out according to description of Wang et al [6].

### Detection of s100a4 and s100a6 expression in different tissues

Three pigs in *H. parasuis *infection group and control group were selected for the analysis of *s100a4 *and *s100a6 *expression in different tissues. Total RNA from 5 porcine organs (inguinal lymph node, heart, spleen, lung, brain) was isolated with RNAprep pure Tissue Kit (TianGen Biotech (Beijing) Co., Ltd). Total RNA was then quantified by NanoDrop 1000 Spectrophotometer (Thermo Fisher Scientific Inc., USA). The quality of the RNA was checked by formaldehyde denaturing gel electrophoresis in 1.2% agarose gels, which showed dispersed bands (28S and 18S) without any obvious smearing patterns that would indicate degradation. Two microgram of total RNA was used for reverse transcription polymerase chain reaction, using the TransSript First Strand cDNA Synthesis SuperMix according to the manufacturer's instructions (TianGen Biotech (Beijing) Co., Ltd). The qPCR assays were performed and analyzed as described above, with primers listed in Table 3.

### Data for STRING and IPA analysis

Differentially expressed (DE) genes were analyzed using STRING http://string.embl.de, a database of known and predicted protein interaction for DE gene encoded proteins. The results were obtained by inputting the list of gene symbol as identifier (organism = *sus scrofa*, combined score = 0.15). *Ppp1r13l *gene was selected for network exploration using Ingenuity Pathway Analysis (Ingenuity^® ^Systems, http://www.ingenuity.com). The data set containing gene identifier and corresponding expression value was uploaded into in the application. The identifier was mapped to its corresponding object in Ingenuity's Knowledge Base; Network Eligible molecules were then overlaid onto a global molecular network so that network of Network Eligible Molecules could be algorithmically generated based on their connectivity.

## Abbreviations

*H. parasuis*: *Haemophilus parasuis*; DE: differentially expressed; FC: fold change; GO: Gene Ontology; QPCR: quantitative real-time PCR; PAM: porcine alveolar macrophages; LPS: lipopolysaccharide; Poly (I:C): polyinosinic acid-polycytidylic acid; PK-15: pig kidney-15; LAMP: loop-mediated isothermal amplification. TSA: trypticase soy agar; TSB: trypticase soy broth; NAD: nicotinamide adenine dinucleotide. MAS: Molecular Annotation System. SAM: Significance Analysis of Microarrays. FDR: false discovery rate. IPA: Ingenuity Pathway Analysis. DAVID: Database for Annotation, Visualization and Integrated Discovery. ORF: open reading frame.

## Competing interests

The authors declare that they have no competing interests.

## Authors' contributions

YW and CL carried out all works in the lab and drafted the manuscript. YF, XL, WL, SL, YL, YL, and CC participated in the animal challenge experiment and immunoassays. CC and JCA participated in the experiment design and coordination. QH conceived the study, and participated in its coordination and helped to draft the manuscript. All authors read and approved the final manuscript.

## Supplementary Material

Additional file 1 **PAMs transcriptome analysis following *H. parasuis *infection using the Affymetrix Porcine Genechip**. Click here for file

Additional file 2 **575 transcripts that are differentially expressed in PAM following *H. parasuis *infection**. Click here for file

Additional file 3 **Categories of annotated DE genes based on biological process GO term. Many categories shared the same transcripts**. Click here for file

Additional file 4 **KEGG Pathway analysis of annotated DE genes**. Click here for file

Additional file 5 **STRING analysis of all annotated DE genes**. Click here for file

Additional file 6 **Phylogenetic tree of coronin 1a**. Click here for file

Additional file 7 **Multiple alignment of the porcine coronin 1a protein with other 8 known coronin 1a proteins**. Click here for file

Additional file 8 **Ppp1r13l related genes by IPA analysis**. Click here for file

Additional file 9 **Primers for nested PCR and LAMP**. Click here for file
