# Supplementary material for: Transcription analysis on response of porcine alveolar macrophages to Haemophilus parasuis
Source: BMC Genomics. 2012 Feb 13;13:68. doi: 10.1186/1471-2164-13-68 (PMC3296652; doi:10.1186/1471-2164-13-68)

**Additional file 5**: STRING analysis of all annotated DE genes. The DE genes were analyzed using the *Sus Scrofa* STRING database. The network nodes represent the proteins encoded by the DE genes. Seven different colored link a number of nodes and represent seven types of evidence used in predicting associations. A red line indicates the presence of fusion evidence; a green line represents neighborhood evidence, a blue line represents coocurrence evidence; a purple line represents experimental evidence; a yellow line represents textmining evidence; a light blue line represents database evidence and a black line represents coexpression evidence.


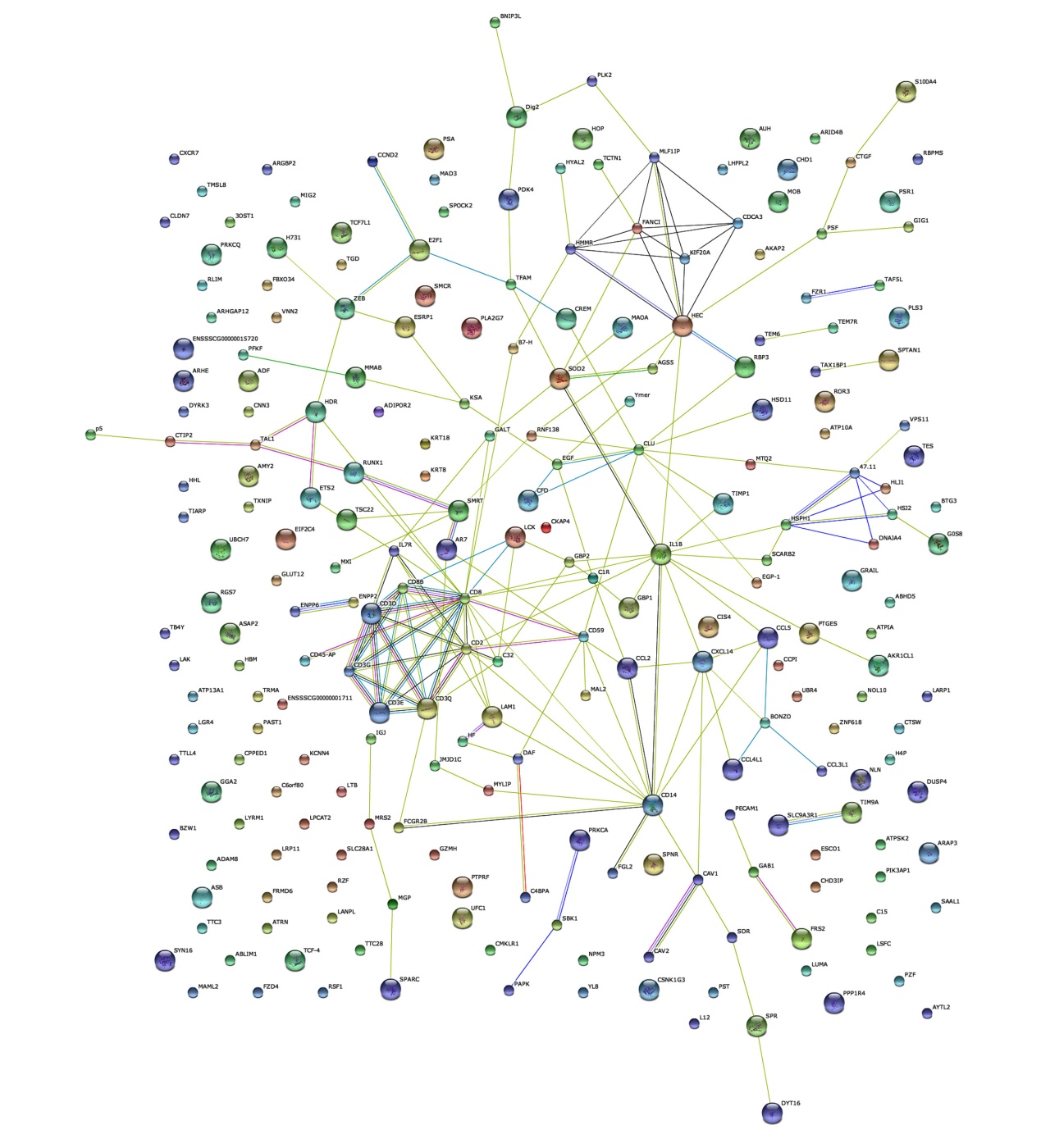

Supplement: Additional file 5 — STRING analysis of all annotated DE genes. [file 1471-2164-13-68-S5.DOC]
