# Supplementary material for: Transcription analysis on response of porcine alveolar macrophages to Haemophilus parasuis
Source: BMC Genomics. 2012 Feb 13;13:68. doi: 10.1186/1471-2164-13-68 (PMC3296652; doi:10.1186/1471-2164-13-68)

**Additional file 6**: Phylogenetic tree of CORONIN 1a. Phylogenetic analysis was conducted using the neighbor-joining method based on multiple alignments of 9 known CORONIN 1a from GenBank using MEGA version 5. The bootstrap confidence values marked at the node of the tree are based on 1000 bootstrap replicates. The horizontal branch lengths are proportional to the estimated divergence of the sequence from the branch point, whereas the vertical branch lengths are arbitrary.


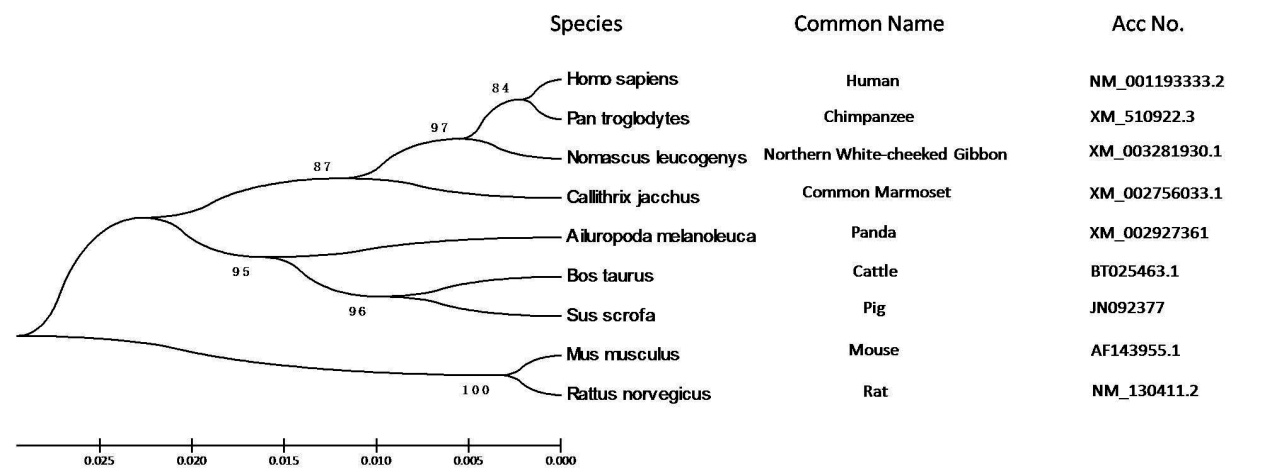

Supplement: Additional file 6 — Phylogenetic tree of coronin 1a. [file 1471-2164-13-68-S6.DOC]
