# Supplementary material for: Transcription analysis on response of porcine alveolar macrophages to Haemophilus parasuis
Source: BMC Genomics. 2012 Feb 13;13:68. doi: 10.1186/1471-2164-13-68 (PMC3296652; doi:10.1186/1471-2164-13-68)

**Additional file 7**: Multiple alignments of the poCORONIN 1A with other 8 known CORONIN 1A proteins. The putative Trp-Asp (WD) repeats signature, Trp-Asp (WD) repeats profile and Trp-Asp (WD) repeats circular profile are indicated by underline. The numbers indicate the aa position.


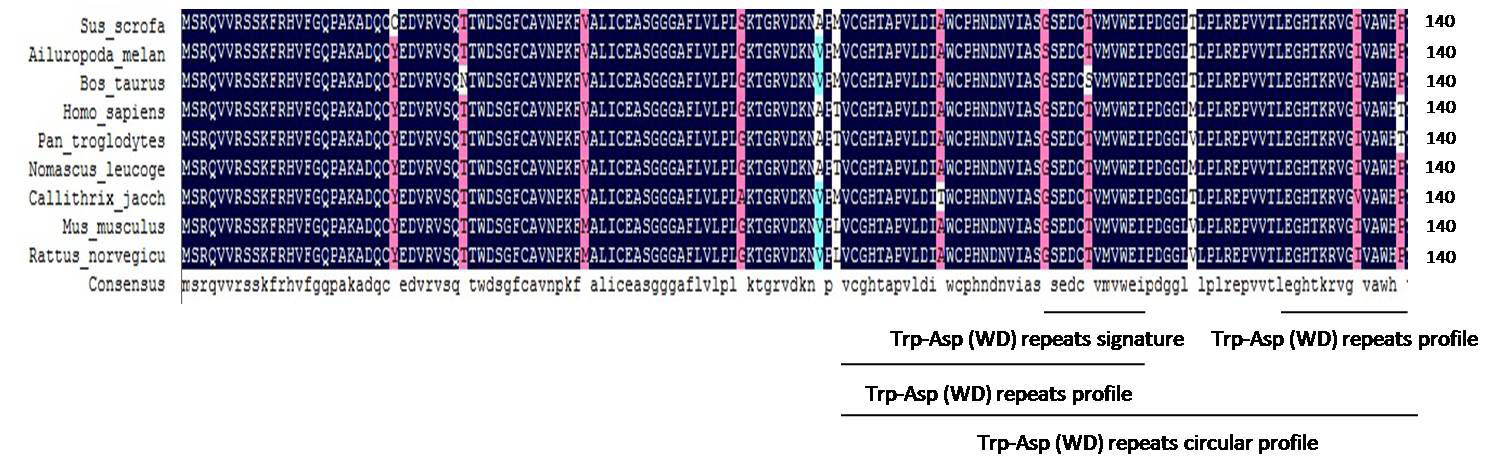


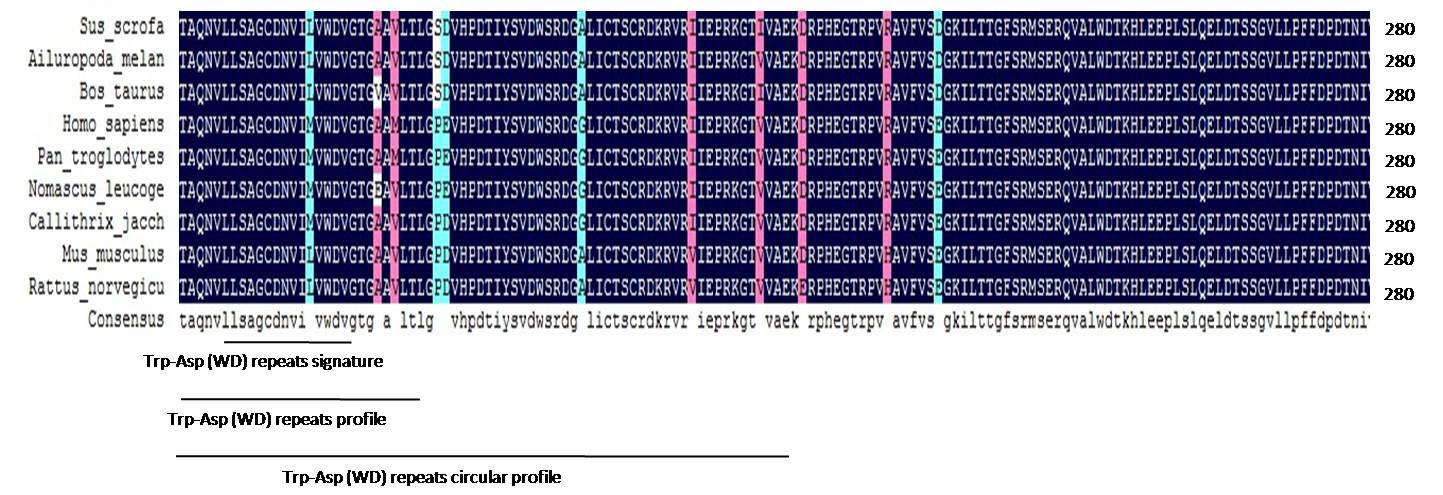


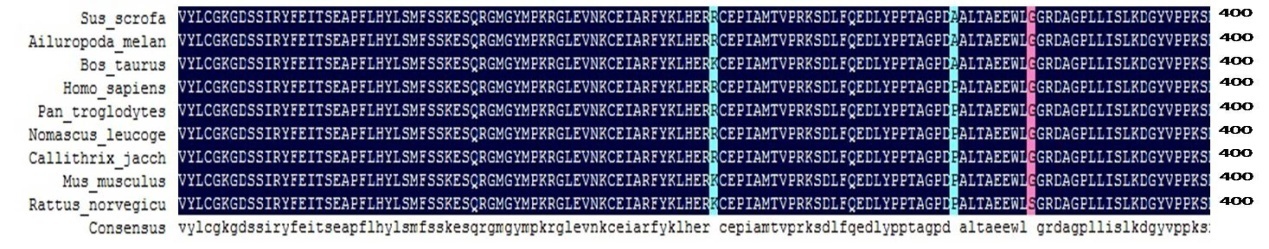


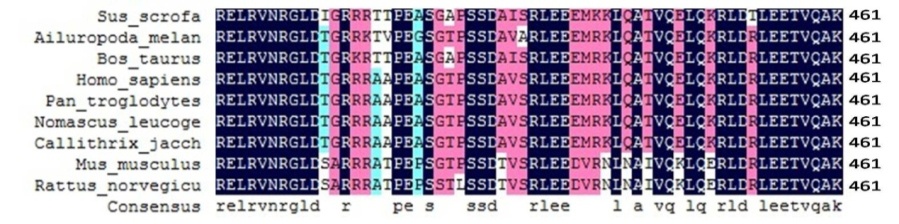

Supplement: Additional file 7 — Multiple alignment of the porcine coronin 1a protein with other 8 known coronin 1a proteins. [file 1471-2164-13-68-S7.DOC]
