# Supplementary figures and images for: Transcription analysis on response of porcine alveolar macrophages to Haemophilus parasuis
Source: BMC Genomics. 2012 Feb 13;13:68. doi: 10.1186/1471-2164-13-68 (PMC3296652; doi:10.1186/1471-2164-13-68)

**Additional file 8**: ppp1r13l related genes by IPA analysis


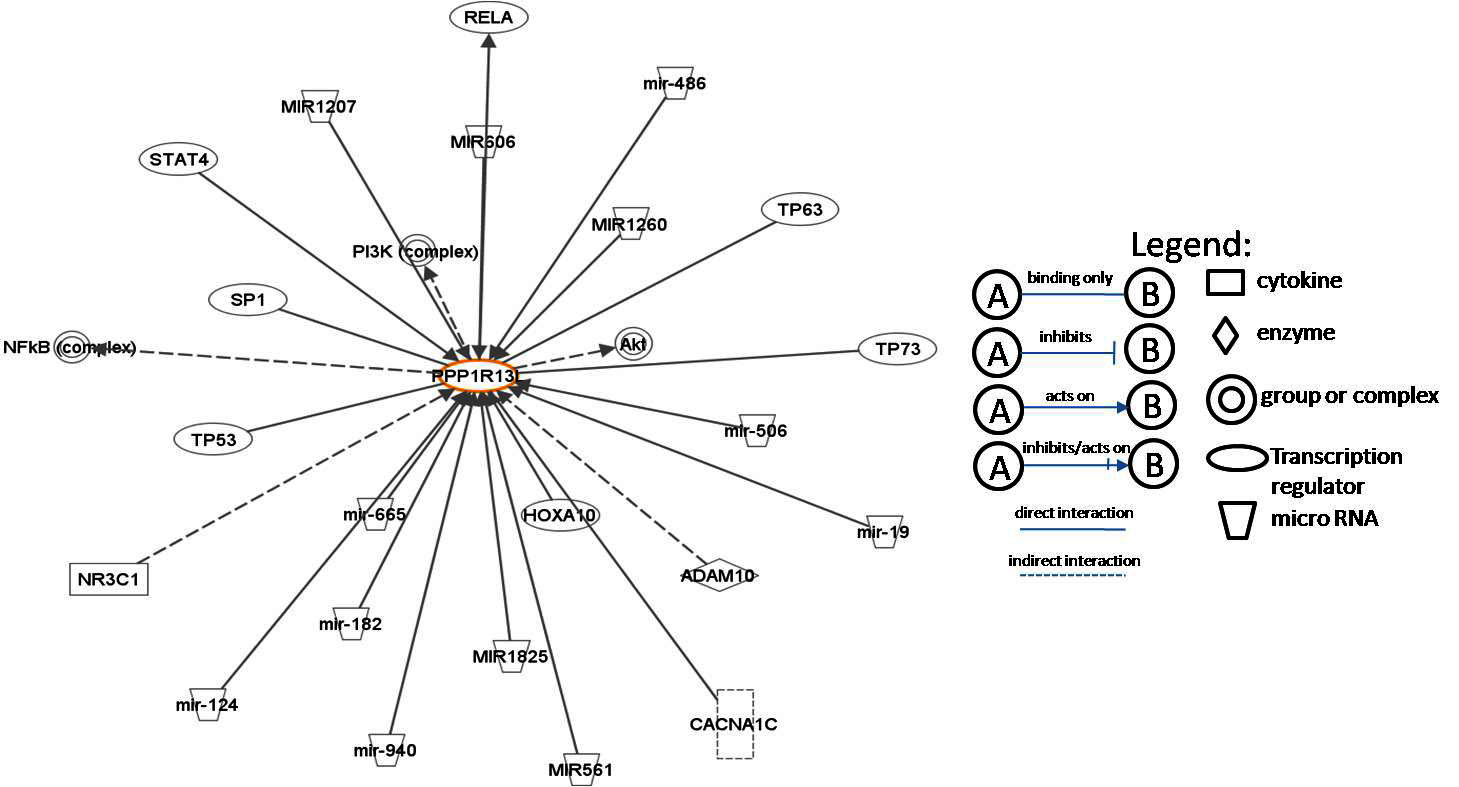

Supplement: Additional file 8 — Ppp1r13l related genes by IPA analysis. [file 1471-2164-13-68-S8.DOC]
