# Supplementary material for: Transcription analysis on response of porcine alveolar macrophages to Haemophilus parasuis
Source: BMC Genomics. 2012 Feb 13;13:68. doi: 10.1186/1471-2164-13-68 (PMC3296652; doi:10.1186/1471-2164-13-68)
Supplement: Additional file 9 — Primers for nested PCR and LAMP. [file 1471-2164-13-68-S9.DOC]

**Additional file 9: Primers for nested PCR and LAMP**

| **Method** | **Primers** | **Sequences** | **Reference** |
| --- | --- | --- | --- |
| **Nested PCR** | HP1F3 | TATCGRGAGATGAAAGAC | Angen 2007 |
|  | HP2F2 | GTAATGTCTAAGGACTAG | Angen 2007 |
|  | HPRevx | CCTCGCGGCTTCGTC | Angen 2007 |
|  | F3 | CGCGGTAATACGGAGGGT | Wang 2010 |
|  | B3 | CTCCACATCTCTACGCATT | Wang 2010 |
| **LAMP** | F3 | CGCGGTAATACGGAGGGT | Wang 2010 |
|  | B3 | CTCCACATCTCTACGCATT | Wang 2010 |
|  | FIP (F1c+TTTT+F2) | GGCTTTCACATCTCACTTAAGTTTTTAGCGTTAATCGGAATGACTG | Wang 2010 |
|  | BIP(B1c+TTTT+B2) | TGCATTTCATACTGGGTTGCTTTTCACCGCTACACGTGGAAT | Wang 2010 |
